# Evaluating the Clinical Impact of Ureteral Frozen Section Analysis During Radical Cystectomy: A Single-Center Retrospective Study

**DOI:** 10.7759/cureus.72908

**Published:** 2024-11-03

**Authors:** Saleh Al-Gburi, Magi Williams, Ketan Agarwal, Thiagarajan Nambirajan

**Affiliations:** 1 Urology, Wirral University Teaching Hospital NHS Foundation Trust, Wirral, GBR

**Keywords:** frozen section, oncologic urology, radical cystectomy, upper urinary tract urothelial carcinoma, urothelial bladder cancer

## Abstract

Introduction

The objective of this study is to find out if there are any differences in upper urinary tract recurrence and overall mortality between patients who underwent a frozen section analysis during radical cystectomy and those who did not.

Materials and methods

In an observational retrospective cohort study, we evaluated data from 164 patients who underwent radical cystectomy in our institution over a five-year period from 2013 to 2018. Fisher's exact test was applied to find any difference in upper urinary tract recurrence between the two groups. The Kaplan-Meier method and the log-rank (Mantel-Cox) test were used to determine differences or equivalence between treatment groups.

Results

The sensitivity was 84.6% and the specificity was 95.3% for the frozen section. There was no statistically significant relationship between performing a frozen section and upper urinary tract recurrence, as indicated by Fisher's exact test (p=0.619). The Kaplan-Meier test showed no statistically significant relationship between performing a frozen section analysis and overall mortality.

Discussion

The use of ureteric frozen section analysis during radical cystectomy is traditionally taught during surgical training, but the evidence base for this practice is sketchy. Frozen section analysis is thought to reduce the chances of local recurrence and arguably upper urinary tract recurrence. The overall upper urinary tract recurrence after radical cystectomy is reported to be 2-6%, consistent with the 3.3% observed in our study.

Conclusion

Our study demonstrates that while frozen section analysis is sensitive and specific in detecting dysplasia, it does not significantly impact upper tract recurrence or overall mortality.

## Introduction

Radical cystectomy (RC) has been the gold standard for muscle-invasive urothelial cancer of the bladder as well as non-muscle-invasive urothelial cancer, with a high risk of progression or that is non-responsive to intra-vesical immunotherapy: Bacillus Calmette-Guérin (BCG) [1]. Muscle-invasive bladder cancer is defined as a tumor that invades the bladder's muscularis propria layer (T2) for which RC combined with urine diversion has been the gold standard treatment for decades [2]. Upper urinary tract recurrence (UUTR) following RC is in the order of 2-8.7% [3,4]. Conventionally, urologists are trained to do frozen section analysis (FSA) at the time of RC to ensure the ureteric margins are clear of cancer. This is thought to reduce the chances of local recurrence and arguably UUTR.

The objective of this study is to find out if there are any differences in UUTR and overall mortality between patients who underwent FSA during RC and those who did not.

## Materials and methods

In an observational retrospective cohort study, we evaluated data from 164 patients who underwent RC in our institution over a five-year period from 2013 to 2018. Basic demographic, clinical, pathological, perioperative, and oncological features were collected retrospectively. The patients underwent open procedures in the early part of the study, and the robotic-assisted laparoscopic approach was introduced in 2015. A full histological examination using hematoxylin and eosin (H&E) stains in permanent sections was performed following FSA. We compared those who had intra-operative FSA of the ureters and those who did not. The sensitivity (SN) and specificity (SP) of FSA were calculated.

Twenty-five patients were excluded as follow-up information was not available. Additionally, 20 patients with a follow-up period of less than 10 months were also excluded from the study due to reasons such as missing records or death from intercurrent illness not related to cancer. The final study cohort comprised 119 patients.

The follow-up assessment included comprehensive medical history, physical examination, and laboratory tests, including full blood count, liver and kidney function tests, urine microscopy, and urine cytology. Computed tomography (CT) scans of the chest, abdomen, and pelvis, with or without the urographic phase, were also performed on these patients depending on the renal function and history of allergy to contrast agents. The follow-up protocol involved appointments every six months for the first two years, followed by annual visits.

Categorical data is presented as frequencies and percentages, while continuous variables are presented as means with standard deviations or medians. Univariate analysis was conducted as follows: continuous variables were compared using either a t-test or a Mann-Whitney test, depending on whether the data were normally or non-normally distributed, respectively. Categorical variables were compared using either Fisher's exact test or the chi-squared test, depending on the specific conditions. The Shapiro-Wilk test was used to assess data normality. Survival curves were generated for each patient group using the Kaplan-Meier method, and the log-rank (Mantel-Cox) test was used to determine differences or equivalence between treatment groups. Patients who did not experience the event were censored at their last follow-up date. Statistical significance was set at p<0.05. All statistical analyses were performed using IBM SPSS Statistics for Windows, Version 25.0 (Released 2017; IBM Corp., Armonk, New York, United States).

## Results

The total number of patients was 164, of which 78 patients had intra-operative FSA of the ureters, while 86 did not.

The definition of true positive is that for those patients who had positive FSA that was confirmed on H&E stain examination, false positive is those patients who had positive FSA that was disproved on H&E stain examination, true negative is those patients who had negative FSA that was confirmed on H&E stain examination, and false negative is those patients who had positive FSA that was disproved on H&E stain examination. The SN was 84.6% and the SP was 95.3% for FSA as illustrated in Table 1.

**Table 1:** The sensitivity and specificity of the frozen section H&E: hematoxylin and eosin; TP: true positive; FN: false negative; FP: false positive; TN: true negative

	Positive on permanent section (paraffin/H&E) (13)	Negative on permanent section (paraffin/H&E) (65)
Positive frozen section (15)	TP (11)	FP (3)
Negative frozen section (63)	FN (2)	TN (62)
SN=(TP/TP+FN)×100=84.6%, SP=(TN/TN+FP)×100=95.3%		

Table 2 illustrates the preoperative demographics and clinic characteristics of the patients who underwent RC after exclusion.

**Table 2:** Preoperative demographics and clinic characteristics ASA: American Society of Anesthesiologists; FSA: frozen section analysis

	FSA (61)	Without FSA (58)	P-value
Gender			
Male (%)	51(84%)	35 (60.3%)	
Female (%)	10 (16%)	23 (39.7%)	
Preoperative creatinine	83 (SD 33)	84 (SD 46)	0.744
ASA 1 (%)	8 (13.1%)	9 (15.5%)	
ASA 2 (%)	46 (77.4%)	29 (50%)	
ASA 3 (%)	7 (11.5%)	20 (34.5%)	
Pre-op chemotherapy			
Yes	12 (19.7%)	12 (20.7%)	0.535
No	49 (80.3%)	46 (79.3)	
Salvage cystectomy	4 (6.6%)	2 (3.4%)	0.154
Open (%)	59 (97%)	41 (70.7%)	0.001
Robotic surgery (%)	2 (3%)	17 (29.3)	

The cancer's pathological features are shown in Table 3.

**Table 3:** Pathological features T0: no evidence of a primary tumor in the bladder; Ta: non-invasive papillary carcinoma

	Frozen section	No frozen section	P-value
T0 (%)	13 (21.3%)	5 (8.6%)	0.167
Ta (%)	4 (6.6%)	2 (3.4%)	
CIS (%)	6 (9.8%)	8 (13.7%)	
T1 (%)	13 (21.3%)	5 (8.6%)	
T2 (%)	7 (11.5%)	11(18.9%)	
T3a (%)	6 (9.8%)	11 (18.9%)	
T3b (%)	8 (13.1%)	14 (24.1%)	
T4 (%)	4 (6.6%)	2 (3.4%)	
N0 (%)	54 (88.5%)	46 (84.5%)	0.232
N1 (%)	6 (9.8%)	6 (10.3%)	
N2 (%)	1 (1.6%)	3 (5.2%)	
R1 (%)	5 (8.2%)	6 (10.3%)	0.154
Squamous differentiation	5 (8.2%)	4 (6.9%)	

We evaluated UUTR in these patients after a median follow-up period of 69 months with a minimum duration of 10 months and a maximum duration of 124 months. The recurrence rates are presented in Table 4.

**Table 4:** UUTR FSA: frozen section analysis; UUTR: upper urinary tract recurrence

	FSA (61)	Without FSA (58)
UUTR	3 (4%)	1 (1.7%)

Among the three patients with UUTR who underwent FSA, one required three FSA before achieving a negative margin, indicating multifocal disease. The other two patients were negative on both FSA and final histological examination. Only one patient without FSA had UUTR but was found to have carcinoma in situ (CIS) in the ureter on final histology.

There was no statistically significant relationship between performing an FSA and UUTR, as indicated by Fisher's exact test (p=0.619).

Regarding the cases with UUTR, two had a T3b stage, one had a T4 stage, and one had CIS on the final histological examination. Additionally, two cases were classified as N1, while the other two were N0. 

**Figure 1:**
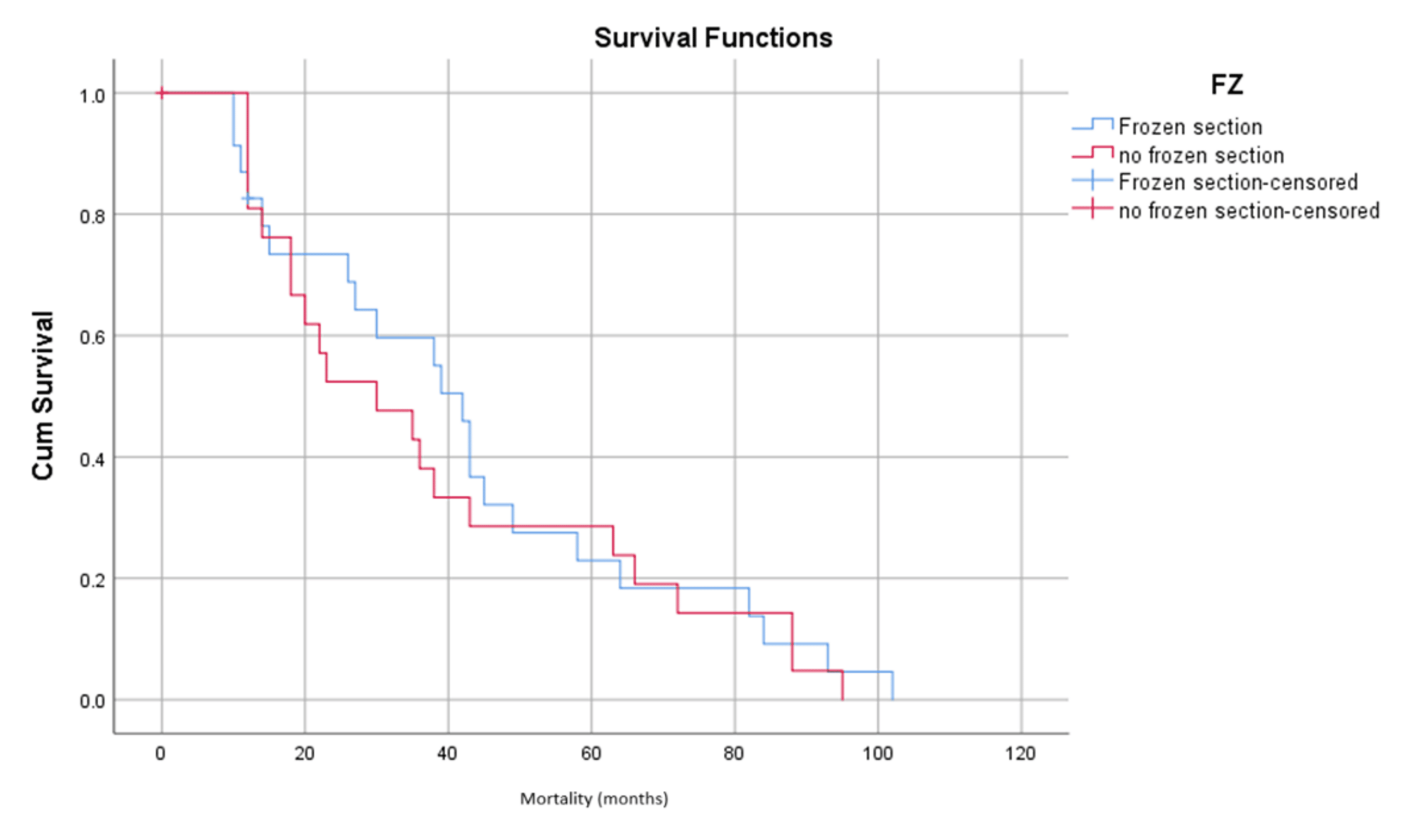
The Kaplan-Meier test

The overall mortality rate was 45 patients (37.8%) over a 35-month period. The Kaplan-Meier test was used to evaluate any differences in survival over time in relation to performing an FSA. The analysis showed no statistically significant relationship between performing an FSA and overall mortality, with a log-rank (Mantel-Cox) value of 0.651, as shown in Figure 1.

## Discussion

The use of ureteric FSA during RC is traditionally taught during surgical training, but the evidence base for this practice is unclear. FSA is thought to reduce the chances of local recurrence and arguably UUTR. The overall UUTR after RC is reported to be 2-6%, consistent with the 3.3% observed in our study [5,6]. Systematic reviews have suggested that UUTR occurs in 4-10% of RC and is associated with poor prognosis and late diagnosis [7]. According to a meta-analysis, patients with non-muscle-invasive bladder cancer are twice as likely to develop UUTR compared to those with invasive disease [8]. Follow-up investigations identified UUTR in 38% of patients, while symptoms led to the diagnosis in 62%. In the current study, all UUTRs were diagnosed by interval imaging.

Some studies advocate FSA routinely during RC, while others do not. Soliman et al. suggested that routine FSA should not be disregarded to achieve tumor-free uretero-enteric anastomosis and reduce the incidence of UUTR [9]. On the other hand, Tang et al. found that even though the ureter was resected until a negative margin was reached, patients with positive ureteric FSA after cystectomy had an increased risk of overall mortality, leading them to suggest omitting FSA during RC for non-muscle-invasive bladder cancer [10].

Mukha et al. noted that ureteral resection to achieve a negative margin on FSA did not appear to prevent upper tract recurrence and concluded that routine FSA may not be necessary for most patients undergoing RC [11]. Similarly, Schumacher et al. stated that if the ureters were resected at the point where they cross the common iliac vessels, ureteric FSA would be unnecessary [12]. The current study did not show any differences in the risk of UUTR between the two cohorts. This led to a change in our practice in our institution to omit routine FSA and resect the ureters at the level of the common iliac artery during RC. We did not find any association between UUTR and non-muscle-invasive bladder cancer, but the number in this study is too small to draw a meaningful conclusion.

No significant factors for UUTR were identified by Rabbani et al. [13]. In our study, however, UUTR occurred in patients with advanced T-stage and lymph node metastasis. It has also been suggested that individuals with intramural or juxtavesical ureter carcinoma involvement or bladder CIS may be at higher risk for UUTR [14,15].

Given that our study found no difference in overall mortality or UUTR between patients who did and did not undergo FSA, it suggests that performing a frozen section until a negative margin is achieved may not impact outcomes and is not recommended.

In our study, the SN and SP of FSA were 84% and 95%, respectively. While the SP aligns with findings from other studies, the SN varies widely across different studies [16,17].

Comparable to previous studies such as Perri et al., who found a 53% mortality rate in 24 months for RC, our study revealed an overall mortality rate of 45 patients (37.8%) over a 35-month period [18]. Kwiatkowska et al. reported that five-year overall survival is expected to be between 50% and 80% based on extensive examination of outcomes following RC [19]. 

The limitations of our study include its retrospective design, single-center setting, and relatively small sample size compared to other studies. However, one of the strengths of our study is the median follow-up period of 69 months. Multicenter studies or systematic reviews of recent literature are strongly advised as they will provide more reliable information.

## Conclusions

Although FSA is sensitive and specific in detecting dysplasia, our data shows that there was no difference in UUTR or mortality between the patients who had FSA and those who did not. Even yet, our study has limitations, as previously mentioned. Future multicenter studies and systemic reviews will be helpful in providing a more definitive conclusion about this topic. 

## References

[1] Cantiello F, Russo GI, Vartolomei MD (2018). Systemic inflammatory markers and oncologic outcomes in patients with high-risk non-muscle-invasive urothelial bladder cancer. Eur Urol Oncol.

[2] Lenis AT, Lec PM, Chamie K (2020). Urinary diversion. JAMA.

[3] Raj GV, Tal R, Vickers A (2006). Significance of intraoperative ureteral evaluation at radical cystectomy for urothelial cancer. Cancer.

[4] Sanderson KM, Cai J, Miranda G, Skinner DG, Stein JP (2007). Upper tract urothelial recurrence following radical cystectomy for transitional cell carcinoma of the bladder: an analysis of 1,069 patients with 10-year followup. J Urol.

[5] Huguet-Pérez J, Palou J, Millán-Rodríguez F, Salvador-Bayarri J, Villavicencio-Mavrich H, Vicente-Rodríguez J (2001). Upper tract transitional cell carcinoma following cystectomy for bladder cancer. Eur Urol.

[6] Balaji KC, McGuire M, Grotas J, Grimaldi G, Russo P (1999). Upper tract recurrences following radical cystectomy: an analysis of prognostic factors, recurrence pattern and stage at presentation. J Urol.

[7] Gakis G, Black PC, Bochner BH, Boorjian SA, Stenzl A, Thalmann GN, Kassouf W (2017). Systematic review on the fate of the remnant urothelium after radical cystectomy. Eur Urol.

[8] Picozzi S, Ricci C, Gaeta M (2012). Upper urinary tract recurrence following radical cystectomy for bladder cancer: a meta-analysis on 13,185 patients. J Urol.

[9] Soliman K, Taha DE, Aboumarzouk OM, Koraiem IO, Shokeir AA (2020). Can frozen-section analysis of ureteric margins at the time of radical cystectomy predict upper tract recurrence?. Arab J Urol.

[10] Tang J, Ranasinghe W, Cheng J (2019). Utility of routine intraoperative ureteral frozen section analysis at radical cystectomy: outcomes from a regional Australian center. Curr Urol.

[11] Mukha R, Kumar S, Kekre NS (2007). Ureteral frozen section analysis during radical cystectomy: do margins matter?. Indian J Urol.

[12] Schumacher MC, Scholz M, Weise ES, Fleischmann A, Thalmann GN, Studer UE (2006). Is there an indication for frozen section examination of the ureteral margins during cystectomy for transitional cell carcinoma of the bladder?. J Urol.

[13] Rabbani F, Perrotti M, Russo P, Herr HW (2001). Upper-tract tumors after an initial diagnosis of bladder cancer: argument for long-term surveillance. J Clin Oncol.

[14] Neuzillet Y, Soulie M, Larre S (2013). Positive surgical margins and their locations in specimens are adverse prognosis features after radical cystectomy in non-metastatic carcinoma invading bladder muscle: results from a nationwide case-control study. BJU Int.

[15] Lee SE, Byun SS, Hong SK (2006). Significance of cancer involvement at the ureteral margin detected on routine frozen section analysis during radical cystectomy. Urol Int.

[16] Touma N, Izawa JI, Abdelhady M, Moussa M, Chin JL (2010). Ureteral frozen sections at the time of radical cystectomy: reliability and clinical implications. Can Urol Assoc J.

[17] Satkunasivam R, Hu B, Metcalfe C (2016). Utility and significance of ureteric frozen section analysis during radical cystectomy. BJU Int.

[18] Perri D, Rocco B, Sighinolfi MC (2024). Open versus robot-assisted radical cystectomy for the treatment of pT4a bladder cancer: comparison of perioperative outcomes. Cancers (Basel).

[19] Kwiatkowska M, Dybowski B, Kuczkiewicz-Siemion O (2017). Factors affecting one-year survival after radical cystectomy: a prospective study. Cent European J Urol.

